# Vitrectomy and scleral imbrication in patients with myopic traction maculopathy and macular hole retinal detachment

**DOI:** 10.1007/s00417-016-3523-7

**Published:** 2016-11-10

**Authors:** Yoshimasa Ando, Akito Hirakata, Arisa Ohara, Reiji Yokota, Tadashi Orihara, Kazunari Hirota, Takashi Koto, Makoto Inoue

**Affiliations:** 1 Kyorin Eye Center, Kyorin University School of Medicine, Tokyo, Japan; 2 Department of Radiology, Kyorin University School of Medicine, Tokyo, Japan

**Keywords:** Optical coherence tomography, three-dimensional magnetic resonance imaging, Scleral imbrication, Macular hole retinal detachment, Myopic traction maculopathy

## Abstract

**Purpose:**

To determine the outcomes of vitrectomy with scleral imbrication in highly myopic eyes with either myopic traction maculopathy (MTM) or macular hole retinal detachment (MHRD).

**Methods:**

The medical records of 17 patients who had undergone vitrectomy with internal limiting membrane (ILM) peeling and scleral imbrication for MTM or MHRD were reviewed. The best-corrected visual acuities (BCVAs), the axial length, the macular hole (MH) closure rate, and the shape of the posterior segment determined by optical coherence tomography were evaluated. Three-dimensional magnetic resonance imaging (3D-MRI) was also performed on five eyes.

**Results:**

The postoperative BCVA improved significantly from 0.76 ± 0.39 logarithm of the minimum angle of resolution (logMAR) units to 0.53 ± 0.35 logMAR units (*P* = 0.0004). The axial length decreased from 29.42 ± 1.81 mm to 27.97 ± 1.71 mm at 1 month. The MTM was resolved or decreased in all eyes. The MH was closed in 44 % of the MHRD eyes, and the retina was reattached in all of the MHRD eyes. The horizontal distance between the optic disc and the bottom of the posterior staphyloma was significantly decreased at 1 month (*P* = 0.012) but not at later times. The 3D-MRI images showed a reduction in the distance between the bottom of the posterior staphyloma and the center of the eye (*P* = 0.029) and a flattening of the posterior staphyloma (*P* = 0.010).

**Conclusions:**

Vitrectomy with ILM peeling and scleral imbrication may be helpful in treating MTM and MHRD by reducing the degree of curvature of the posterior staphyloma.

## Introduction

Pathologic myopia is associated with a reduction of visual acuity due to chorioretinal abnormalities including myopic choroidal neovascularization, lacquer cracks, patchy or diffuse chorioretinal atrophy, and pigmentary degeneration [1, 2]. The axial lengths of highly myopic eyes are abnormally long, and the posterior surface of the eye is enlarged posteriorly [3–5]. Consequently, the retina and choroid are stretched and thinned leading to the development of myopic traction maculopathy (MTM) [6–9].

Optical coherence tomography (OCT) has shown that MTM usually begins with macular retinoschisis followed by the development of a foveal detachment and a subsequent macular hole retinal detachment (MHRD) [10, 11].

Vitrectomy with internal limiting membrane (ILM) peeling has been used to treat MTM and MHRD [12–15]. The postoperative complications after treatments for MTM, such as a macular hole (MH) and MHRD, tend to worsen the visual outcomes [13, 16]. Recent case series studies showed that vitrectomy with ILM peeling for MHRD achieved a retinal reattachment rate ranging from 42 to 100 % [14, 15, 17].

Matsumura and Ogino [18] used vitrectomy with scleral resection and gas tamponade to successfully treat eyes with MHRD. Matsuo and associates [19] simplified this technique by using scleral imbrication instead of scleral resection, and they reported on the effects of vitrectomy combined with scleral imbrication and gas tamponade in two eyes that had a reopening of a macular hole and three eyes that had a persistent retinal detachment after an initial vitrectomy for MHRD. The macular hole was closed and the retina reattached in all eyes. However, the pathology on how the scleral shortening by lamellar resection or imbrication affected the highly myopic eyes and the long-term surgical outcomes have not been determined.

Thus, the purpose of this study was to assess anatomical and visual outcomes of vitrectomy with scleral imbrication in highly myopic eyes with MTM and MHRD, and also to evaluate the changes of the curvature of the posterior surface of the eyes by OCT and three-dimensional magnetic resonance imaging (3D-MRI) before and after the surgery.

## Methods

The medical records of 17 highly myopic eyes of 17 patients (1 man and 16 women) who underwent vitrectomy and scleral imbrication for MTM or MHRD were studied. The surgery was performed because of a progressive decrease of vision due to the MTM or the MHRD. The eyes were treated between June 2012 and July 2014 at the Kyorin Eye Center. High myopia was defined as a refractive error greater than −6.0 diopters (D) or axial length ≥26 mm [1]. Seven eyes were pseudophakic preoperatively with an axial length ≥26 mm. Three patients had an initial surgery at another hospital and were referred to our clinic for further treatments. The patients were followed for at least 6 months postoperatively.

The mean age of all patients was 69.8 ± 7.5 years with a range of 58 to 78 years, and the mean follow-up period was 15.1 ± 9.8 months with a range of 6 to 38 months. All surgeries were performed after the patients received a detailed explanation of the surgical and OCT procedures. Informed consent was obtained from all patients, and the procedures adhered to the tenets of the Declaration of Helsinki. The study protocol was approved by the Institutional Review Board of the Kyorin University School of Medicine. All patients consented to the review and use of their medical records for future research studies. This clinical study has been registered at the United States National Institutes of Health (www.clinicaltirals.gov) as “Vitrectomy and Scleral Shortening for Macular Hole Retinal Detachment or Myopic Traction Maculopathy” with a reference number of NCT02528045.

The patients underwent standard ophthalmological examinations that included stereoscopic fundus observations, measurements of the best-corrected visual acuity (BCVA) and axial length, and optical coherence tomography (OCT) pre- and postoperatively. The clinical demographics and the BCVA [logarithm of the minimal angle of resolution (logMAR) units] were evaluated. Spectral-domain OCT (Spectralis, Heidelberg Engineering, Heidelberg, Germany) or swept-source OCT (DRI-OCT1, Topcon, Tokyo, Japan) was used for the OCT examinations. The axial length was measured by OA1000 (TOMEY, Japan).

The surgery was performed by one of the authors (AH) at the Kyorin Eye Center. A 23- or 25-guage vitrectomy system (Alcon Constellation^@^ Vision System, Alcon Laboratories, Fort Worth, TX, USA) with a cutting rate of 5000 cuts/min and aspiration pressure of 650 mmHg was used. A retrobulbar injection of 2 % lidocaine was given for anesthesia. One case (case 10) underwent surgery under general anesthesia because the patient had difficulty in maintaining an appropriate position. The lens was extracted from all patients if the eye was phakic followed by phacoemulsification with simultaneous implantation of an intraocular lens in four eyes or secondary implantation in six eyes. Core vitrectomy was performed with the creation of a posterior vitreous detachment (PVD) in all eyes. An intravitreal injection of triamcinolone acetonide (MaQaid®, Wakamoto Pharmaceutical Co., Ltd., Tokyo, Japan) was used to make the transparent vitreous gel more visible. The ILM was peeled within the vascular arcade after an intravitreal injection of brilliant blue G (BBG) to make the ILM more visible in all cases.

Then, four to five mattress sutures of 5-0 polyester suture (Dacron, Alcon Laboratories, Fort Worth, TX) were preplaced with 6–9-mm bite widths of the sutures. They were placed in the superotemporal and inferotemporal quadrants at the equatorial sclera. The preplaced mattress sutures were tightened to imbricate the temporal sclera after the infusion pressure was lowered. Then, 20 % SF6 gas or silicone oil was injected to complete the surgery. Four patients had a secondary implantation of a toric intraocular lens after resolution of the MTM and reattachment of MHRD to correct for postoperative astigmatism after scleral imbrication and vitrectomy.

**Fig. 1:**
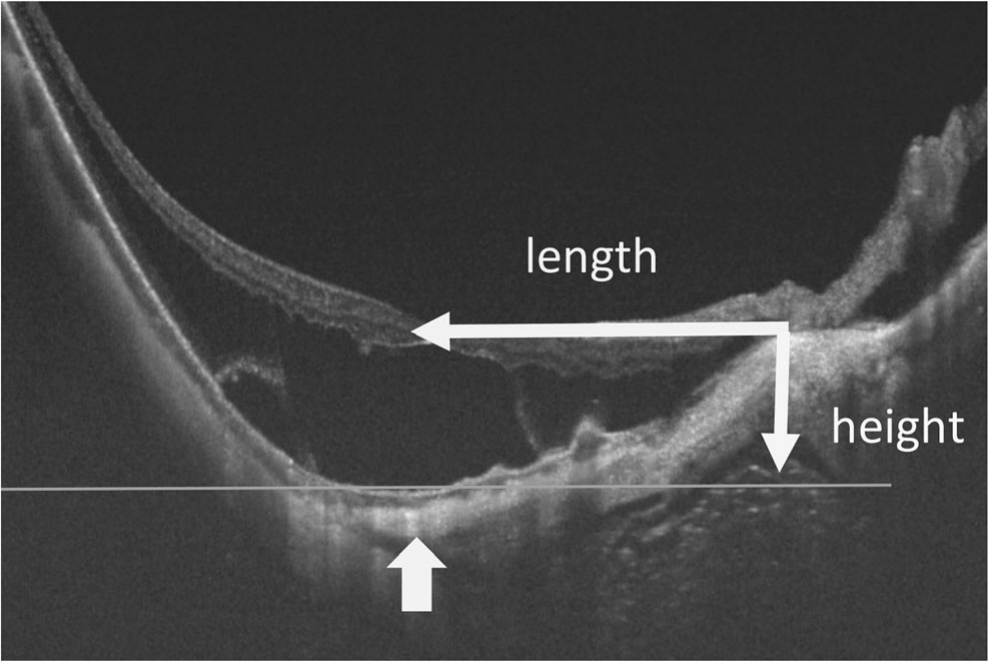
Horizontal optical coherence tomography (OCT) image showing how the different parameters of a posterior staphyloma and optic disc were measured. The height and length of the staphyloma are defined as the horizontal and vertical lengths from the temporal edge of the optic disc to the plane of the bottom of the retinal pigment epithelial layer, respectively

To evaluate the posterior curvature of the eye before and after the scleral imbrication, horizontal spectral-domain OCT or swept-source OCT scans were recorded with the eye in the primary position (Fig. 1). The horizontal and vertical distances from the temporal edge of the optic disc to a plane at the bottom of the retinal pigment epithelium layer were defined as the length and height. These measurements were made preoperatively and postoperatively at 1, 3, and 6 months (*n* = 8).

**Fig. 2:**
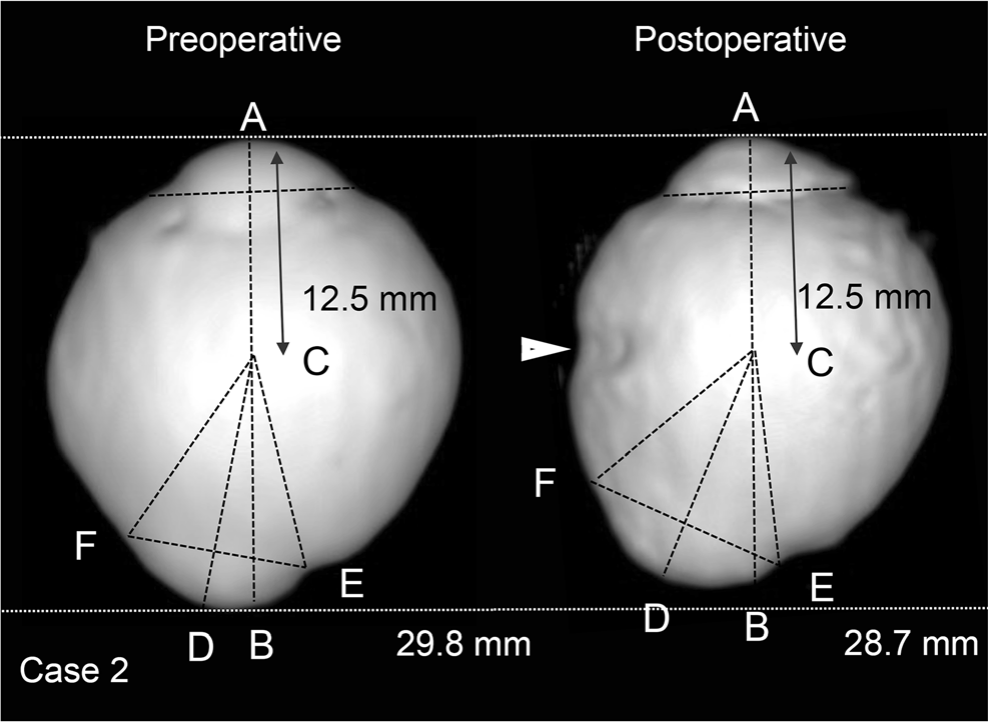
Preoperative and postoperative three-dimensional magnetic resonance images of case 2. The corneal apex (*A*) is defined as the point farthest from the plane of the corneal limbus. The central axes (*AB line*) of the eye is defined as a line that intersects the plane of the corneal limbus perpendicularly. The center (*C*) of the eye is selected to be 12.5 mm posterior from the peak of the cornea along the central axis. *D*. The bottom of the posterior staphyloma is defined as the point farthest from the center of the eye within the staphyloma. Indicated are the nasal edge (*E*), temporal edge (*F*), and width (*EF*) of the posterior staphyloma. The postoperative image shows an infolding of the eye globe by the scleral imbrication (*white arrowhead*) and the decrease of the axial length of the eye

Three-dimensional magnetic resonance imaging (3D-MRI; Signa HDxt 1.5T, GE Healthcare, UK) was performed before and after surgery in five patients (Fig. 2). To evaluate the 3D-MRI findings in eyes that had scleral imbrication, the center of the eye was defined by a modified method reported by Moriyama and associates [20, 21]. The central axis of the eye was selected to cross the plane of the corneal limbus perpendicularly. The center of the eye was selected to be 12.5 mm posterior to the peak of the cornea along the central axis. The angle from the central line of the eye to the temporal edge and the nasal edge of the posterior staphyloma, the length of central line behind the center of the eye (posterior axial length), the length of the bottom of the staphyloma (the farthest point from the center of the eye within the staphyloma) from the center of the eye, and the width of the staphyloma between the temporal and nasal edges were evaluated in the horizontal OCT images.

## Results

The mean age (± standard deviation) of all patients was 69.8 ± 7.5 years with a range of 58 to 78 years, and the mean follow-up period was 15.1 ± 9.8 months with a range of 6 to 38 months. All of the eyes were pseudophakic at the final examination. The retinal detachment in eyes with MHRD was found in the temporal quadrants in four eyes and was within or around the vascular arcade in five eyes. A gas tamponade was used in 13 eyes, air in 1 eye, and SF6 in 12 eyes. Silicone oil was injected into four eyes of four patients because these patients had difficulty maintaining a prone position or had poor vision in the fellow eye.

**Fig. 3:**
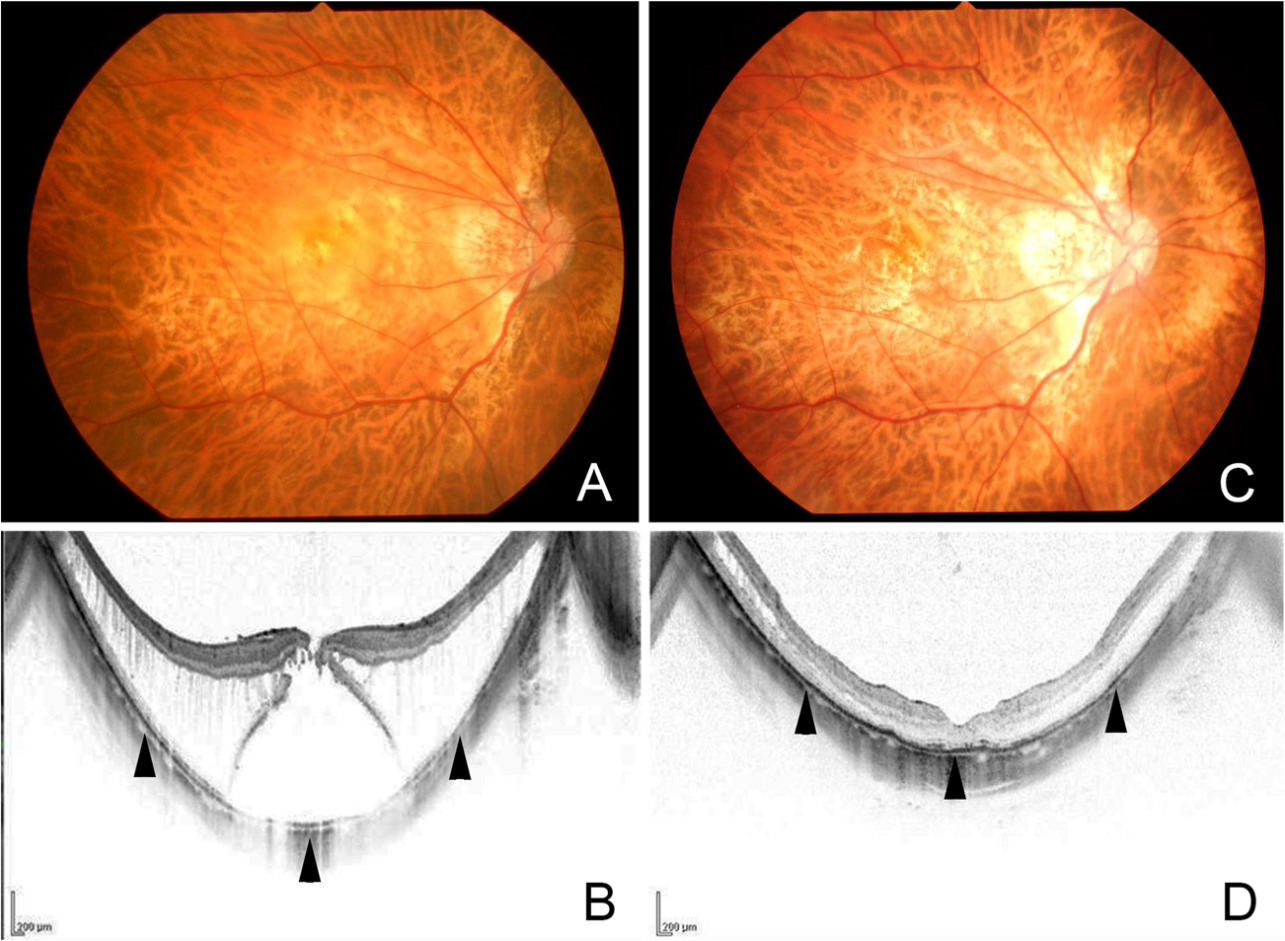
Preoperative and postoperative fundus photographs and optical coherence tomography (OCT) images of case 2. **a**. Preoperative fundus photograph and (**b**) OCT image showing macular retinoschisis and foveal detachment. The posterior surface (*arrowheads*) is extruded posteriorly due to a posterior staphyloma. **c**. Postoperative photograph and (**d**) OCT at 6 months shows a resolution of the macular retinoschisis and foveal detachment. The posterior surface (*arrowheads*) is flattened postoperatively

**Fig. 4:**
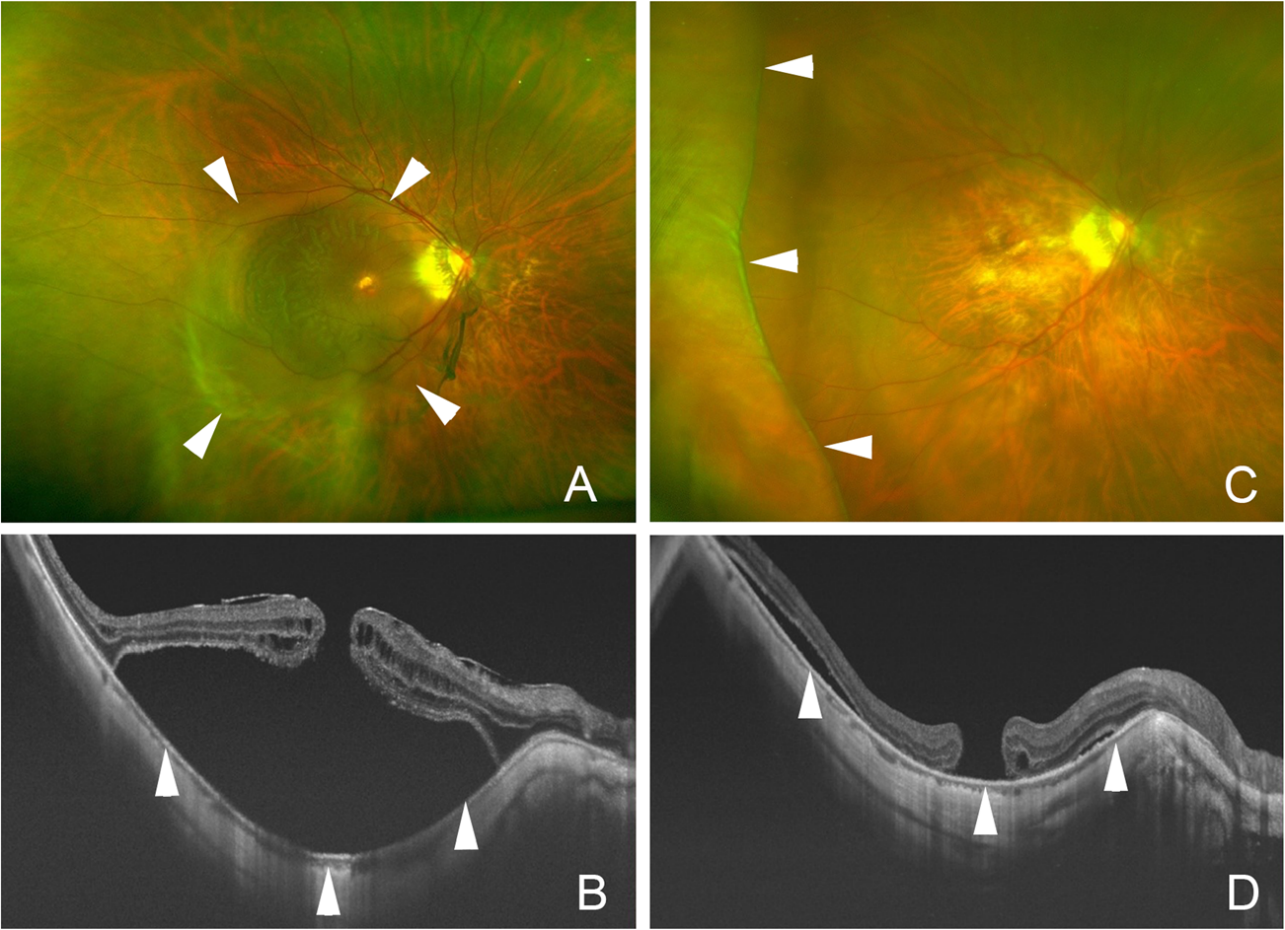
Preoperative and postoperative ultra-wide-field fundus images and optical coherence tomography (OCT) images of case 8. **a**. Preoperative ultra-wide-field fundus image and (**b**) OCT image show the macular hole retinal detachment (*arrowheads*). The posterior surface (*arrowheads*) is extruded posteriorly due to a posterior staphlyoma. **c**. Postoperative ultra-wide-field fundus image at 6 months showing retinal attachment and scleral infolding (*arrowheads*) at the temporal quadrant. **d**. Postoperative OCT image shows retinal reattachment with residual subretinal fluid. The posterior curvature (*arrowheads*) is flatter postoperatively

A resolution of the MTM or a decrease of the macular retinoschisis was achieved in all eyes with MTM, and none of the eyes with a foveal detachment and macular retinoschisis developed a MH postoperatively (Table 1). The retina was reattached in all eyes with a MHRD (Figs. 3 and 4), and the MH was closed in 4 of 9 eyes (44 %).

**Table 1:** Characteristics and anatomical outcome of the subjects with myopic traction maculopathy

Case	Diagnosis	Preop. lens status	Preop. AL (mm)	Byte width (mm) and number of stitches (×)	Shortened AL (mm)*	Attached macula	MH closure	Follow-up duration (month)
1	MHRD	IOL	26.00	7 mm × 5	1.79	+	+	17
2	MHRD	Phakia	29.83	9 mm × 4	1.59	+	+	38
3	MHRD	Phakia	29.42	9 mm × 4	2.16	+	−	6
4	MHRD	IOL	30.96	6 mm × 4	0.97	+	+	15
5	MHRD	Phakia	30.82	7 mm × 5	2.59	+	+	21
6	MHRD	Phakia	30.53	8 mm × 5	0.35	+	−	6
7	MHRD	IOL	27.30	9 mm × 4	0.88	+	−	25
8	MHRD	Phakia	28.59	8 mm × 5	0.75	+	−	13
9	MHRD	Phakia	29.43	8 mm × 5	2.00	+	−	6
10	FD	IOL	30.53	9 mm × 4	0.35	+	NA	6
11	FD	IOL	26.25	7 mm × 5	0.73	+	NA	27
12	FD	Phakia	30.15	8 mm × 5	2.97	+	NA	14
13	FD	IOL	26.23	8 mm × 5	0.43	+	NA	6
14	MH, FD	Phakia	29.95	6 mm × 4	0.98	+	−	26
15	MR	Phakia	32.04	8 mm × 5	2.26	decreased	NA	6
16	MR	Phakia	27.51	7 mm × 5	2.02	decreased	NA	19
17	MR	IOL	31.41	6 mm × 5	1.96	decreased	NA	6

*MHRD* macular hole retinal detachment, *FD* foveal detachment, *MR* macular retinoschisis, *preop* preoperative, *IOL* intraocular lens, *AL* axial length, *NA* not applicable, * = shortened axial length at postoperative 1 month compared with preoperative length, decrease = attached macula with decrease of macular retinoschisis

**Fig. 5:**
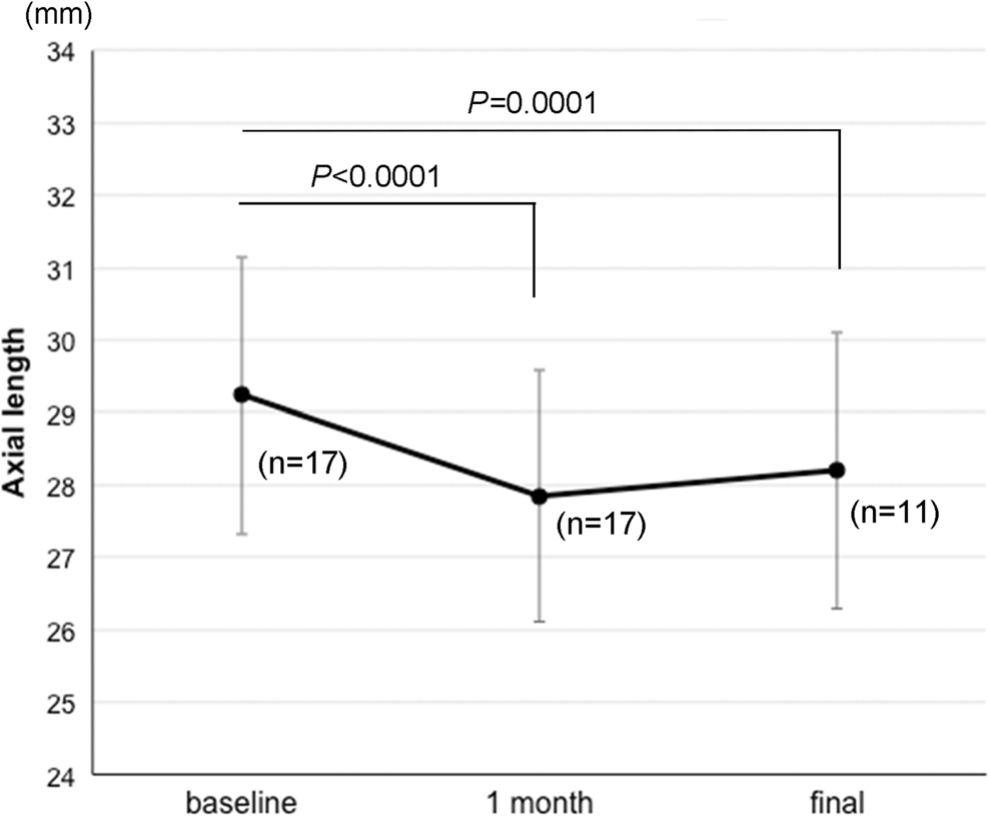
The mean axial length at baseline and at 1 month and the final visit after scleral imbrication. The mean axial length is decreased significantly at 1 month and the final visit from the baseline (Wilcoxon signed-rank test)

**Fig. 6:**
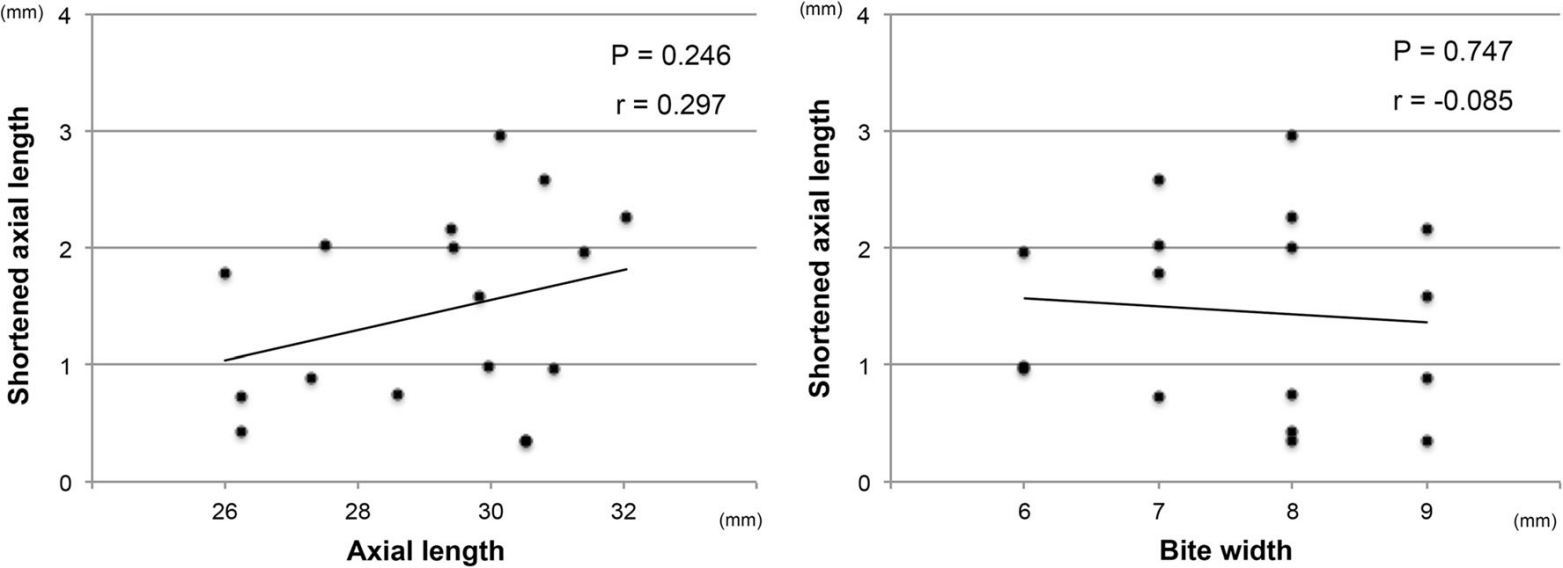
The correlation between the shortened axial length and the bite width of the mattress suture for scleral imbrication. The shortened axial length from the baseline is not significantly correlated with the baseline axial length and the bite width of the mattress suture for scleral imbrication

The mean preoperative BCVA was 0.76 ± 0.39 logMAR units which improved significantly to 0.53 ± 0.35 logMAR at 6 months postoperatively (*P* = 0.0004, Wilcoxon signed-rank test). The mean axial length was 29.4 ± 1.8 mm with a range of 26.0 to 32.0 mm prior to scleral imbrication, and it decreased significantly to 28.0 ± 1.7 mm (*P* < 0.0001, Wilcoxon signed-rank test) with a range of 25.2 to 30.2 mm at 1 month following the scleral imbrication (Fig. 5). The axial length at the final visit of 20.1 ± 8.7 months postoperatively was 28.2 ± 1.9 mm which was significantly shorter than that of the baseline (*P* = 0.0001). The shortened axial length after the scleral imbrication was not correlated with the baseline axial length and with the bite width of the mattress sutures (Fig. 6). The postoperative refraction was shifted by +1.7 ± 0.7 D (range from +0.9 to +2.5 D) of the predicted refraction in the four eyes that underwent simultaneous cataract surgery with intraocular lens implantation in combination with vitrectomy and scleral imbrication.

### Evaluation of posterior staphyloma with OCT findings

**Fig. 7:**
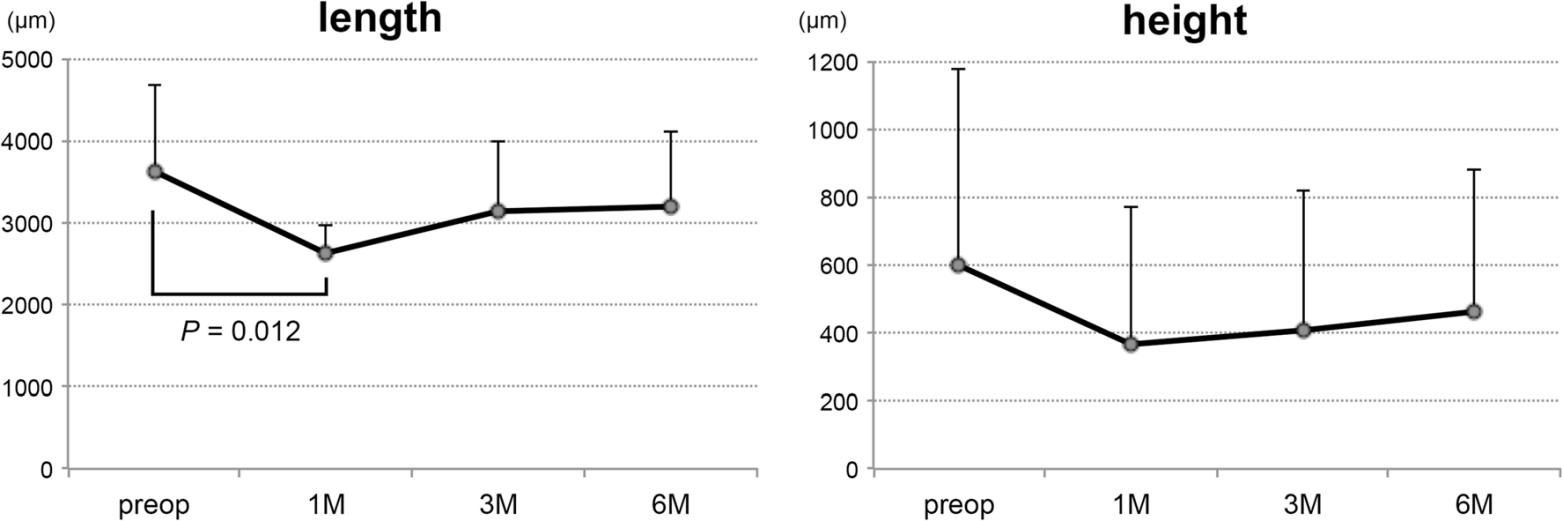
The change of the shape of the posterior staphyloma. The length of the bottom of the posterior staphyloma from the optic disc decreases significantly at 1 month but those at 3 and 6 months are not significant. The height of the bottom of the posterior staphyloma from the optic disc decreases postoperatively but the decrease is not significant

The OCT images showed that the curvature of the posterior staphyloma became less curved, i.e., a longer radius of curvature, after the surgery accompanied by a resolution of the MTM (Figs. 3 and 4). The distance from the bottom of the posterior staphyloma to the level of the optic disc decreased significantly at 1 month but the decreases at 3 and 6 months were not significantly different (Fig. 7, *n* = 8). The height of the bottom of the posterior staphyloma from the optic disc decreased postoperatively but the decrease was not significant at 1, 3, and 6 months. The eye with a staphyloma located in the peripapillary area (case 9) was not evaluated because the optic disc was higher than the bottom of the staphyloma. Preoperative OCT images of the retinal pigment epithelium that were used to determine the bottom of the staphyloma and the optic disc in a single image were not obtained in eight eyes because of the limitation of the scanning depth of the OCT and the presence of bullous retinal detachment. These nine eyes were excluded from the analyses.

### Evaluation of eye shape and posterior staphyloma with 3D-MRI findings

**Fig. 8:**
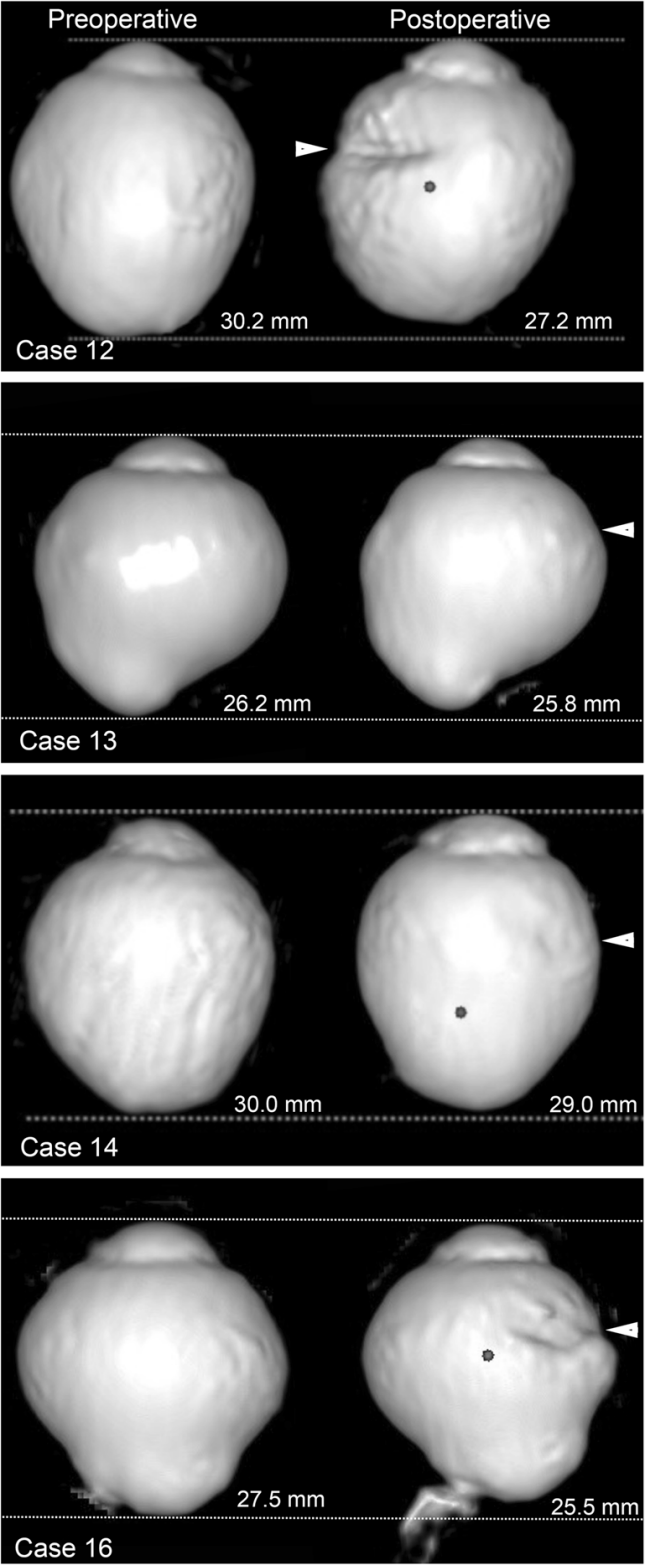
Preoperative and postoperative three-dimensional magnetic resonance imaging (3D-MRI) images. 3D-MRI images show posterior extrusion of the eye preoperatively in all eyes. The postoperative images show the infolding caused by the scleral imbrication (*white arrowheads*) and the decrease of the axial length of the eye. The curvature of the posterior surface of the staphyloma became less extruded posteriorly after surgery

The 3D-MRI images showed changes in the shape of the globe (Fig. 8). The value of each parameter used to evaluate the eye shape is shown in Table 2. The posterior axial length (BC; Fig. 2) and the depth of staphyloma (CD) decreased significantly, and the width of staphyloma (EF) increased significantly. These changes indicated that the depth of posterior staphyloma had decreased and the staphyloma was flatter after the scleral imbrication. There was also a decrease in the axial length. Significant increases of the angle corresponding to the area of staphyloma from the center (BCF and ECF; Fig. 2) also indicated a flattening of the posterior staphyloma.

**Table 2:** The parameters of eyeball shape in three-dimensional magnetic resonance imaging before and after surgery

Case	BC	CD	EF	∠BCD	∠BCE	∠BCF	∠ECF
Preop.							
2	4.8	5.0	4.0	15	13	43	56
12	5.2	5.2	2.6	6	13	16	29
13	4.0	4.2	3.5	44	11	45	56
14	4.8	4.8	4.0	11	36	19	55
16	4.3	4.4	3.9	14	27	31	58
Postop.							
2	4.3	4.7	4.2	30	6	56	62
12	4.5	4.5	3.5	9	19	26	45
13	3.3	4.0	4.0	67	7	67	74
14	4.7	4.7	4.8	15	29	40	69
16	3.8	3.9	4.4	19	26	59	85
*P* value*	0.010	0.029	0.010	0.063	0.345	0.004	0.009

*Preop*. preoperative, *postop*. postoperative, * ; paired *t* test

## Discussion

Nakagawa and associates [22] evaluated the changes in the axial length of eyes after scleral lamellar resection of 6 to 10-mm width or imbrication of 10-mm width in non-highly myopic eye bank eyes. The average shortening of the axial length following lamellar resections of 6 to 10 mm was 1.50 to 2.65 mm, and that for a 10-mm imbrication was 2.50 mm. The differences in the axial lengths before and after scleral shortening were significantly different for the dissections of different amounts and not significantly different between the 10-mm resection and imbrication. However, these eyes were not highly myopic eyes and the average axial lengths were 24.38 mm in the resection group and 24.57 mm in the imbrication group.

We found a decrease of the axial length following scleral imbrication in highly myopic eyes with MTM or MHRD. However, the shortening of the axial length was 1.4 mm at one month after the scleral imbrication which is less than that reported by Nakagawa and associates on normally sized eyes [22]. The anatomical reattachment may not be related to the shortening of the axial length after vitrectomy and scleral imbrication for MTM and MHRD. The retina was also reattached in five eyes with MHRD that was located within or around the vascular arcade. Thus, we believe that the retinal reattachment was related to the changes in the curvature of the posterior staphyloma because the effect of the shortening of the axial length may be limited in eyes without a peripheral retinal detachment.

OCT analysis of the changes in the curvature of a posterior staphyloma showed that the bottom of the staphyloma was raised by the scleral imbrication in the temporal quadrant with an increase in the horizontal distance between the bottom of the staphyloma and the optic disc, and a decrease in the vertical distance between the bottom of the staphyloma and the optic disc. However, there is a limitation in this OCT study of scleral imbrication because the fixation point at the fovea also appeared to shift toward the temporal quadrant, and the scanning direction of the OCT scans were not identical before and after the scleral imbrication.

We also evaluated the shape of the eye in the 3D-MRI images. The 3D-MRI images showed a flattening of the posterior staphyloma following scleral imbrication which is compatible with the evaluation of the posterior staphyloma by OCT. Moriyama and associates [21] classified the shapes of highly myopic eyes by the 3D-MRI into four types based on an inferior view; temporally distorted, nasally distorted, cylindrical, and barrel-shaped. They analyzed the symmetry and pointedness of the posterior segment of the highly myopic eyes. In their study, 36.7 % of the patients had the nasally distorted type and 16.7 % had the temporally distorted type. They also reported that the horizontal asymmetry and the pointedness of the posterior curvature of highly myopic eyes might be important factors that cause the development of most of the vision-threatening complications specific to pathologic myopia. All of the eyes we evaluated by 3D-MRI appeared to be the temporally distorted type, and the temporal distortion and the pointedness appeared to decrease after scleral imbrication. However, the effect of scleral imbrication may differ according to the original shape of the eyes.

Episcleral macular buckling with a solid silicone plate designed for macular indentation can also alter the degree of posterior extrusion of a staphyloma [23]. A combination of vitrectomy and episcleral macular buckling has been reported to lead to better anatomical outcomes in eyes with retinal reattachment compared to vitrectomy alone [23–25]. Parolini and associates [26] reported on an L-shaped macular buckle for MHRD and macular retinoschisis in highly myopic eyes. The retina was attached by vitrectomy and macular buckle in 100 % of the 10 eyes with an MHRD and the macular hole was closed in 60 %. In the five eyes with an MHRD treated with a macular buckle alone, the retina was reattached in five eyes and the macular hole was closed in three eyes. The axial length was shortened by 1.21 mm and the MRI showed a flattening of the posterior staphyloma. We believe that the advantage of sclera imbrication is that it does not require surgical procedures around the macula. In contrast, Hoang and associates [27] reported that the curvature radius of the posterior sclera was reduced after a spontaneous resolution of a macular retinoschisis in highly myopic eyes. They assumed that the decrease of the tractional force from the ILM, vitreous cortex, and staphyloma may be related to the reduced curvature radius of the posterior sclera. Thus, vitrectomy alone may have released the vitreous traction leading to the increased curvature of the posterior sclera.

A closure of the MH was found in 4 (44 %) of 9 eyes with MHRD as confirmed by EDI-OCT or swept-source OCT. Fujikawa and associates [28] performed temporal scleral imbrication combined with PPV, ILM peeling, and gas tamponade in 8 eyes with an MHRD and achieved reattachment in all eyes and MH closure in 6 of the 8 eyes (75 %) with a mean axial length of 29.5 ± 1.3 mm. They had a higher rate of MH closure than our 44 % with a mean axial length of 29.2 ± 1.7 mm; however, the shape of the posterior staphyloma may have affected the rate of MH closure. In addition, a postoperative MH did not develop in any of the eyes with MTM. One of the advantages of our technique is that it can prevent the development of a MH after surgery in eyes with MTM.

This study has several limitations. This was a retrospective, non-comparative case series conducted in a single academic hospital, and the number of patients was small. Therefore, further studies with larger sample sizes are needed to confirm these results.

In conclusion, the results showed that vitrectomy and scleral imbrication can be a treatment option that can lead to successful retinal attachment for eyes with MTM or MHRD. A MH closure in patients with MHRD and the prevention of a MH after surgery for MTM can be achieved by vitrectomy and scleral imbrication in association with reduction of the posterior staphyloma curvature.
